# Two-Dimensional Carbon Film-Supported ZnS Nanocomposites Obtained from Thermal Decomposition of Organic Zinc Salts and Sulfidation Reactions for Lithium Storage

**DOI:** 10.3390/molecules30040893

**Published:** 2025-02-14

**Authors:** Denghu Wei, Ting Wang, Ranran Jiao, Lixue Qu, Suyuan Zeng

**Affiliations:** 1 School of Materials Science and Engineering, Liaocheng University, Liaocheng 252059, China; wangtinglcu@163.com (T.W.); 19861210661@163.com (L.Q.); 2 School of Chemistry and Chemical Engineering, Liaocheng University, Liaocheng 252059, China; jrr1175@mail.ustc.edu.cn

**Keywords:** ZnS/C, carbon film, one-step reaction, lithium storage, general method

## Abstract

This article reports a general method for carbon-composited ZnS nanoparticles. By mixing thiourea with an appropriate amount of citric acid zinc, glycine zinc, lactate zinc, and gluconate zinc, respectively, and then heating at 700 °C under a nitrogen atmosphere for 4 h, four types of target product ZnS/C were obtained. Thiourea and organic zinc salts serve as reactants, providing zinc, sulfur, and carbon sources. During the thermal decomposition process, sulfidation and carbonization can be completed simultaneously. As an anode material for lithium-ion batteries, all four products exhibit excellent lithium storage performance. The two-dimensional carbon film can, on the one hand, enhance the conductivity of the material, and on the other hand, act as a carrier for ZnS particles, effectively cushioning the volume deformation of ZnS during the lithiation process.

## 1. Introduction

Compared with metal oxides, metal sulfides generally have better conductivity, and the band gap energy of M-S bonds is smaller, which is more easily broken in electrochemical reactions. Therefore, metal sulfides as anode materials for lithium-ion batteries have better electrochemical reactivity [1,2,3,4,5,6,7,8]. Among them, zinc sulfide (ZnS) has attracted widespread attention from researchers (not only in the field of lithium-ion batteries [9,10,11,12,13,14,15,16], but also in the fields of sodium-ion [17,18,19,20], potassium-ion [21], lithium-sulfur batteries [22,23,24], and supercapacitors [25,26]) due to its abundant reserves, environmental friendliness, and high capacity. ZnS stores lithium based on conversion and alloying reaction mechanisms, which can cause significant volume expansion and contraction during the cycling process. The stress generated by this change can lead to the pulverization and collapse of the electrode structure, resulting in rapid capacity decay. Nanosizing and carbon composites are effective methods to alleviate this problem [9,10,11,12,13]. When the size of the electrode material is reduced to the nanoscale, the specific surface area increases, the contact area between the electrode and the electrolyte increases, and the diffusion distance of lithium ions in the electrode material is significantly reduced. Moreover, nanosizing can reduce the stress generated during the intercalation and deintercalation process of lithium ions, thereby improving the lithium storage performance of the material. In addition, the composite of zinc sulfide and carbon can improve the conductivity of the electrode material and alleviate the volume expansion and contraction during the cycling process (because amorphous carbon has a certain flexibility), thereby improving the material’s cycle stability.

Based on the above analysis, we selected thiourea (primarily providing a sulfur source, and also possibly providing a portion of the carbon source) and lactate zinc (zinc source, carbon source) as reactants and carried out thermal decomposition and sulfidation (synchronously completed) under a nitrogen atmosphere to prepare carbon composite ZnS nanostructures. This composite acts as an anode material for lithium-ion batteries and shows high capacity and cycle stability. We have expanded this method to prepare three other types of carbon composite ZnS nanomaterials, and all have been tested for lithium storage performance, with good results.

## 2. Results and Discussion

**Figure 1:**
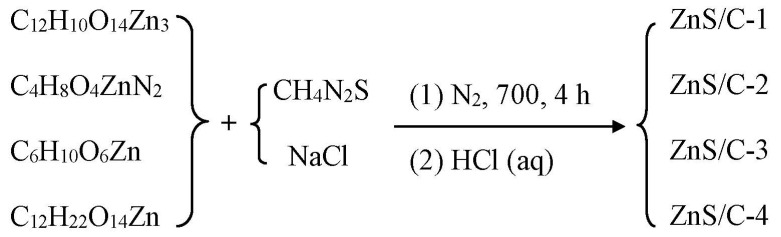
Synthetic route to prepare Zn/C samples.

**Figure 2:**
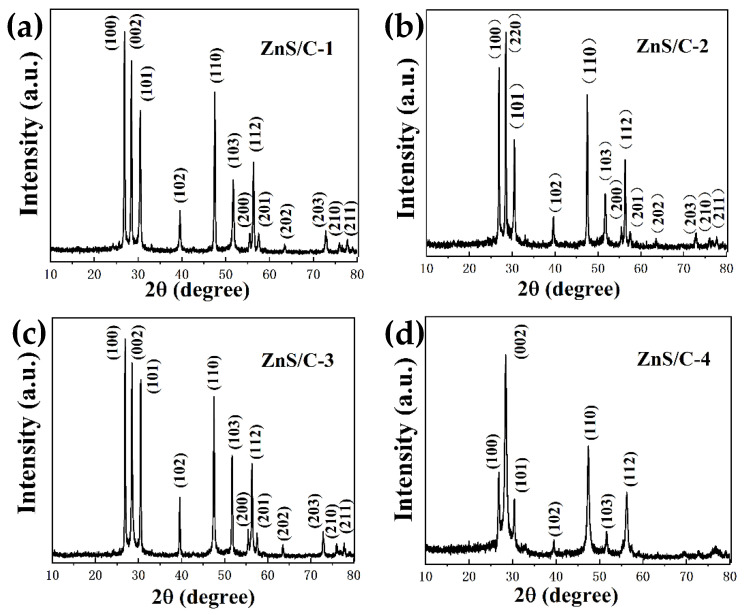
XRD of the samples: (**a**) ZnS/C-1; (**b**) ZnS/C-2; (**c**) ZnS/C-3; and (**d**) ZnS/C-4.

**Figure 3:**
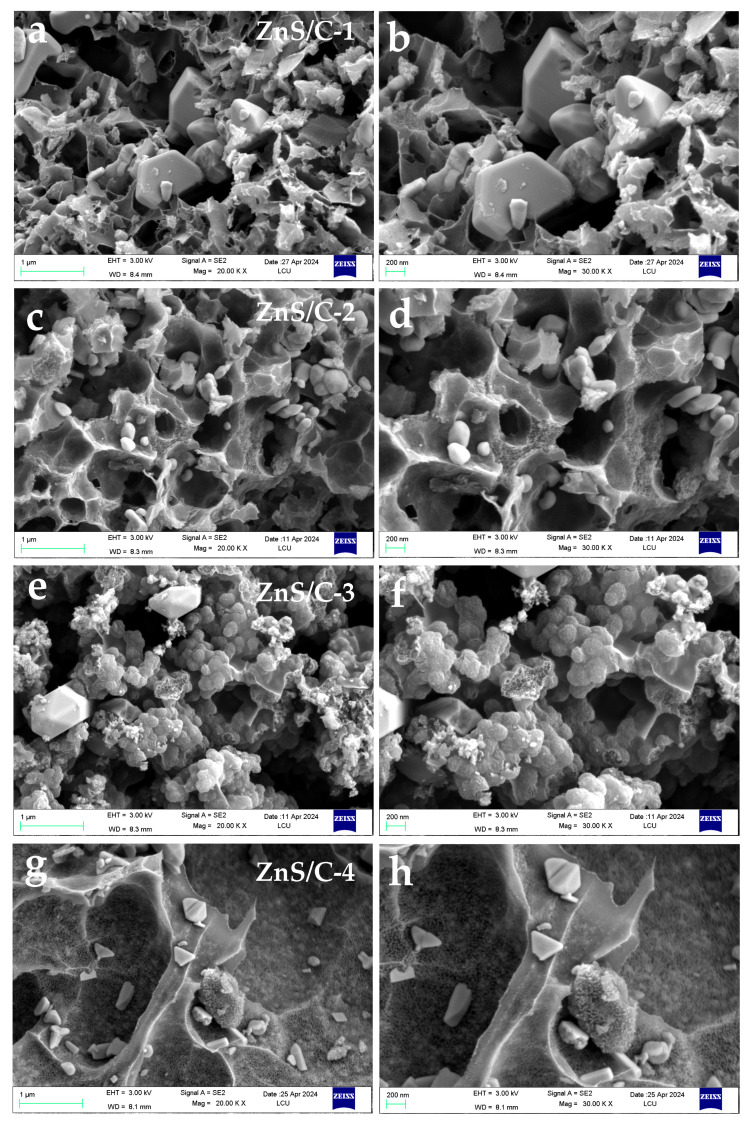
SEM images of the samples: (**a**,**b**) ZnS/C-1; (**c**,**d**) ZnS/C-2; (**e**,**f**) ZnS/C-3; and (**g**,**h**) ZnS/C-4.

**Figure 4:**
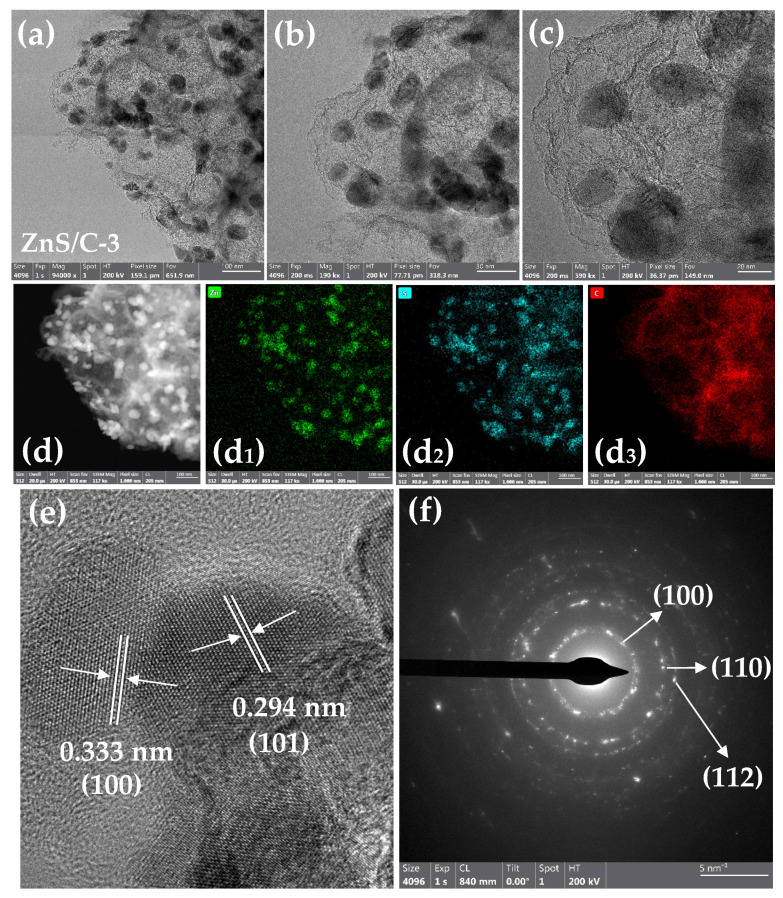
TEM images (**a**–**c**), EDS mapping (**d**–**d3**), HRTEM image (**e**), and electron diffraction (**f**) of the ZnS/C-3 sample.

Figure 1 is a schematic diagram of the synthesis of ZnS/C. Organic zinc salts served as zinc and carbon sources; thiourea mainly served as a sulfur source and may also have provided some carbon source; sodium chloride acted as a heat transfer medium, a load body for the reactants, and a template for pore formation. During the heat treatment under a nitrogen atmosphere, carbonization and sulfidation could be completed synchronously, resulting in the target product. After pyrolysis, the black product was washed with diluted hydrochloric acid to remove impurities such as sodium chloride, resulting in various ZnS/C products. Figure 2 is the XRD pattern of ZnS/C. It can be seen from the figure that all the characteristic peaks correspond to the diffraction peaks of hexagonal ZnS crystals (PDF# 79-2204). The diffraction peak of carbon is not obvious, indicating that the carbon produced is amorphous. Figure 3 and Figures S1–S4 are the SEM morphologies of the products. It can be clearly seen from the figure that in the ZnS/C-3 sample, ZnS and carbon are the best composite, that is, ZnS nanoparticles (about 100 nanometers) are coated in the carbon film. The image formed by the transmission electron microscope also confirms this situation, as shown in Figure 4a–c. In the other three types of ZnS/C samples, some ZnS particles are hidden in the carbon skeleton, and some particles are attached to the surface of the carbon layer. The overall composite effect is not as good as that of ZnS/C-3. The subsequent lithium storage performance test also confirmed this, that is, the performance of ZnS/C-3 is superior.

**Figure 5:**
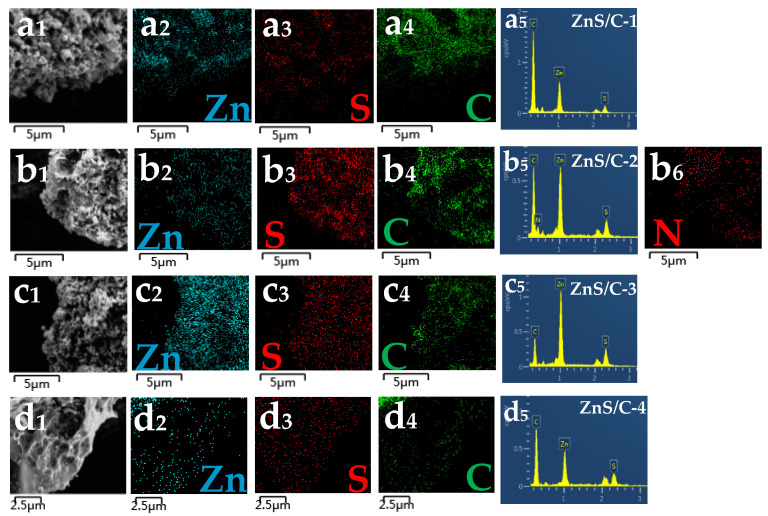
EDS spectra and mapping of the samples: (**a_1_**–**a_5_**) ZnS/C-1; (**b_1_**–**b_6_**) ZnS/C-2; (**c_1_**–**c_5_**) ZnS/C-3; and (**d_1_**–**d_5_**) ZnS/C-4.

**Figure 6:**
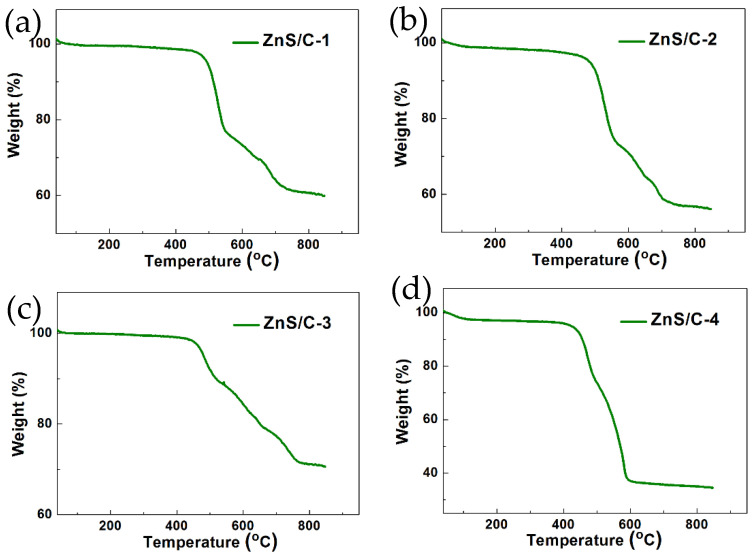
TG curves of the ZnS/C materials under air atmosphere: (**a**) ZnS/C-1; (**b**) ZnS/C-2; (**c**) ZnS/C-3; and (**d**) ZnS/C-4.

Figure 4d–d3 show the elemental distribution mapping of ZnS/C-3. The results show that the distribution of various elements in the sample is relatively uniform. Figure 4e and Figure S5 are HRTEM images, from which more crystallographic information about the ZnS/C-3 sample can be obtained. The measured interplanar spacings in the figure are 0.333 nm and 0.294 nm, corresponding to the (100) and (101) planes of hexagonal ZnS crystals, respectively. The electron diffraction results (Figure 4f) further confirm the crystal structure of ZnS/C-3. Figure 5 shows the EDS spectra and elemental distribution of the four types of ZnS/C samples. The results show that various elements are well distributed in their respective samples, appearing in their respective EDS spectra. To identify the carbon content in the ZnS/C products, we performed thermogravimetric analysis tests on the four samples (Figure 6). Under an air atmosphere, the product would undergo the following reaction:

ZnS/C + O_2_ = ZnO + SO_2_ + CO_x_ (1)

According to the reaction formula (1), the carbon content in each sample can be calculated, which is 27% (ZnS/C-1), 31.7% (ZnS/C-2), 13.8% (ZnS/C-3), and 55.6% (ZnS/C-4), respectively. The carbon content in sample No.4 (ZnS/C-4) is the highest because its reactant is gluconate zinc, and the molar ratio of carbon to zinc in the gluconate zinc is the largest, which is 12.

**Figure 7:**
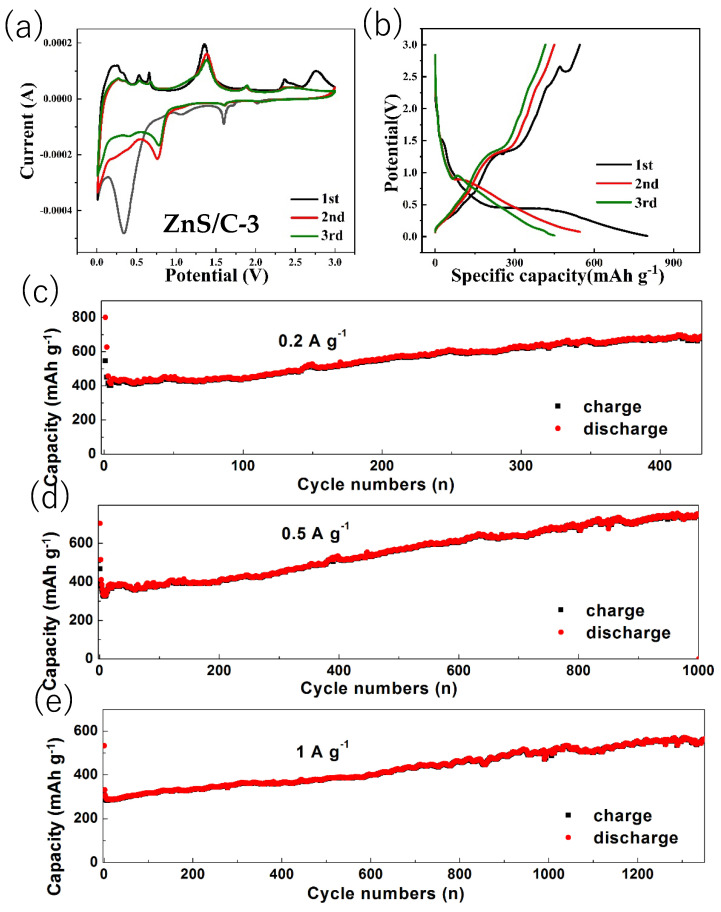
CV curves (**a**), charge–discharge profiles (**b**), and cycle performance (**c**–**e**) of the ZnS/C-3.

Figure 7a shows the first three CV curves of ZnS/C-3, with a voltage range of 0.01 to 3 V and a scan rate of 0.2 mV s^−1^. During the first scan, a reduction peak is located near 0.4 V, corresponding to the conversion reaction of ZnS, generating Li_2_S and elemental zinc [9,10,11]. The peak near 0.01 V corresponds to the alloying reaction of lithium with zinc, as well as the formation of the solid-state electrolyte interphase (SEI) film. In the initial anodic scan, the weak peaks between 0.2 and 0.7 V represent the delithiation process of the lithium-zinc alloy [10,11,12]. Several peaks between 1.2 and 3 volts are attributed to the transformation of zinc to zinc sulfide [11,12,13,14]. In the subsequent two CV curves, the position of the reduction peak shifts to the right due to the activation of the electrode after the first cycle. Figure 7b shows the first three charge–discharge curves of ZnS/C-3, with a current density of 0.2 A g^−1^ and a voltage range consistent with the CV test. The initial discharge capacity is 800 mAh g^−1^, and the charge capacity is 546 mAh g^−1^, with a corresponding initial Coulomb efficiency (CE) of 68%. The loss of capacity in the first cycle is mainly due to the formation of the SEI film, causing irreversible lithium ion extraction [1,2,3,4,5,6,7,8,9]. From the fourth cycle, the CE remains above 95%, indicating that after three activation cycles, the ZnS/C-3 electrode exhibits good reversibility. Figure 7c–e show the cycling performance test results of ZnS/C-3 at current densities of 0.2 A, 0.5 A, and 1 A g^−1^, respectively. The results show that under the three different current densities, the capacity of the ZnS/C-3 electrode will go through a process of initial decline followed by a gradual increase. The initial capacity decay during cycling may be due to the insufficient contact between the particles inside ZnS and the electrolyte, which cannot fully demonstrate their lithium storage capacity. As the charge and discharge cycles continue, the battery capacity gradually increases, indicating that the internal particles of the electrode material are gradually activated and gradually exhibit lithium storage performance. Of course, the specific reasons for the capacity increase during the cycle need further research to confirm. After cycling for 1000 cycles at a current density of 0.5 A g^−1^, the ZnS/C-3 electrode can still maintain a discharge capacity of 749 mAh g^−1^. The good lithium storage performance of ZnS/C-3 under different current densities is mainly due to the following: the ZnS particles are almost fully encapsulated in the carbon film during the formation process (Figure 3e,f and Figure 4), which can effectively prevent particle aggregation during the charge and discharge process; the carbon film with certain flexibility can buffer volume deformation; and although the ZnS particles will still have problems such as pulverization and aggregation during the cycle, the changed particles can still be attached to the carbon film, avoiding loss from the electrode. Compared with the literature reports, the ZnS/C-3 material shows certain improvements in terms of cycle numbers or capacity retention rate, as shown in Table S1. Moreover, the synthesis method is simple: by selecting an organic zinc salt that is homologous with carbon and reacting it with thiourea, the carbonization process and the formation of the zinc sulfide phase can be simultaneously achieved. This method is universal, and when other types of organic zinc sources are used, carbon composite zinc sulfide materials can still be obtained (ZnS/C-1, ZnS/C-2, and ZnS/C-4), and the lithium storage performance of the resulting materials is also quite excellent (Please refer to the section below).

**Figure 8:**
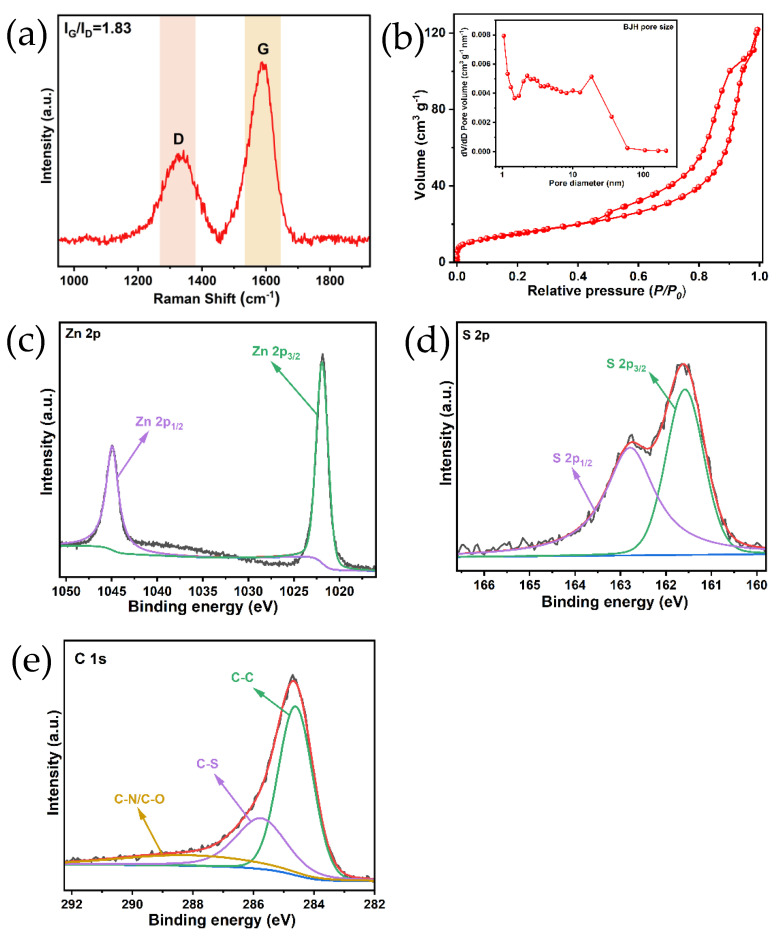
Raman spectrum (**a**), N_2_ adsorption–desorption and pore size distribution diagrams (**b**) of ZnS/C-3. XPS spectra of Zn 2p (**c**), S 2p (**d**), and C 1s (**e**) of the ZnS/C-3 sample.

To further understand the excellent lithium storage performance of ZnS/C-3, we conducted additional characterizations on this product, such as Raman spectroscopy (Figure 8a), specific surface area testing (Figure 8b), and X-ray photoelectron spectroscopy (XPS) analysis (Figure 8c–e). Raman spectroscopy is a very powerful tool to verify the presence of carbon in the composite and the degree of graphitization. As shown in Figure 8a, the I_G_/I_D_ ratio based on the area under the D- and G-band was 1.83, implying that a portion of the carbon component in the ZnS/C-3 sample had been graphitized, which helped to enhance the electronic conductivity of the sample when used as an anode material for lithium storage [9]. Figure 8b shows the nitrogen adsorption–desorption isotherms and the BJH pore size distribution curve (the inset) of the ZnS/C-3 sample. The measured data indicate that the specific surface area of the sample is 54.541 m^2^/g (a*_s, BET_*), and the average diameter of pores is 10.85 nm (based on the BJH model analysis). This pore structure is beneficial for the electrode material to be fully wetted by the electrolyte, and it may also increase some active sites, thereby enhancing the lithium storage performance of the material [9,11]. Figure 8c shows the XPS spectrum of Zn 2p, with two peaks located at 1021.95 eV and 1044.95 eV, corresponding to Zn 2p_3/2_ and Zn 2p_1/2_, respectively, thereby confirming the presence of Zn ions (Zn^2+^) [9]. It is worth noting that the binding energy values of these peaks are slightly higher than those of Zn ions in pure ZnS, indicating a strong interaction between ZnS particles and the carbon film (C-Zn-S-C), which facilitates electron transfer between ZnS particles and the carbon film [12,19]. Figure 8d shows two peaks at 161.6 eV and 162.8 eV, confirming the presence of sulfur ions (S^2−^). Figure 8e presents the XPS spectrum of C 1s, further revealing the existence of C-S bonds (at 285.8 eV), which is consistent with Figure 8c.

**Figure 9:**
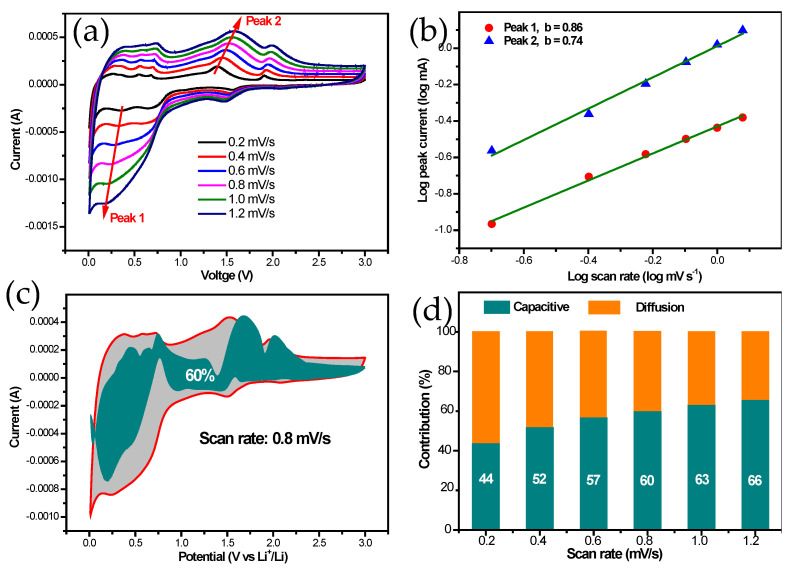
(**a**) CV curves at various scanning rates, (**b**) Linear relation between log (peak current) and log (scan rate), (**c**) surface capacitance contributions (dark cyan) at 0.8 mV s^−1^ and (**d**) the percentage of surface capacitance contributions at various scanning rates of the ZnS/C-3.

To understand the mechanism of charge storage in the ZnS/C-3 electrode, the electrochemical kinetic behavior was investigated via CV curves with different scan rates. Figure 9a shows the CV curves at different scan rates. It can be observed that the shapes of the CV curves are basically similar at different scan rates, and the peak current is directly proportional to the scan rate. With the increase in scan rate, the peak positions of the CV curves shift, indicating that the electrode material undergoes a certain degree of polarization [9,11,12]. Figure 9b shows the fitted curves of log(i) versus log(v). Calculations reveal that for the ZnS/C-3 electrode, the b values corresponding to the oxidation peak and reduction peak are 0.86 and 0.74, respectively (both values lie between 0.5 and (1)). This suggests that the electrochemical reactions of the electrode involve both diffusion-controlled behavior and capacitive behavior [9,11,12]. As the scan rate increases, the contribution of capacitance increases (Figure 9c,d), which is beneficial for the cycling stability of the electrode material [9,11,12]. This also explains the excellent lithium storage performance of ZnS/C-3 (Figure 7c–e).

**Figure 10:**
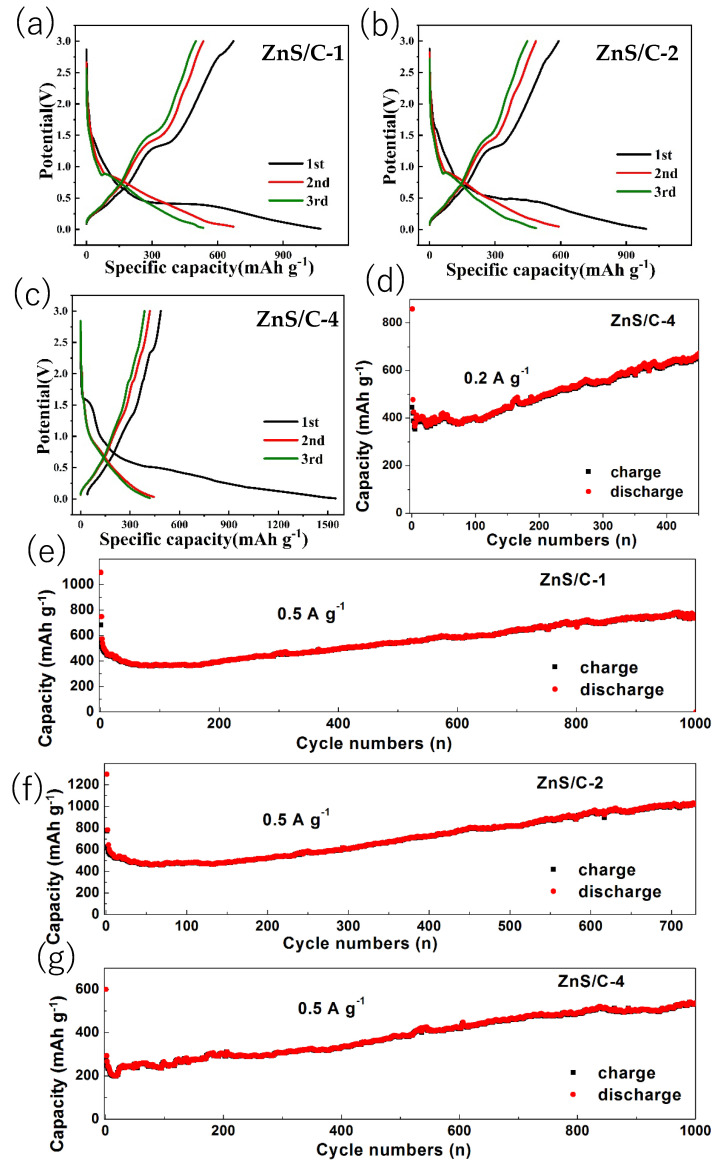
Charge–discharge profiles and cycle performances of the ZnS/C anode materials: (**a**,**e**) ZnS/C-1; (**b**,**f**) ZnS/C-2; (**c**,**d**,**g**) ZnS/C-4.

**Figure 11:**
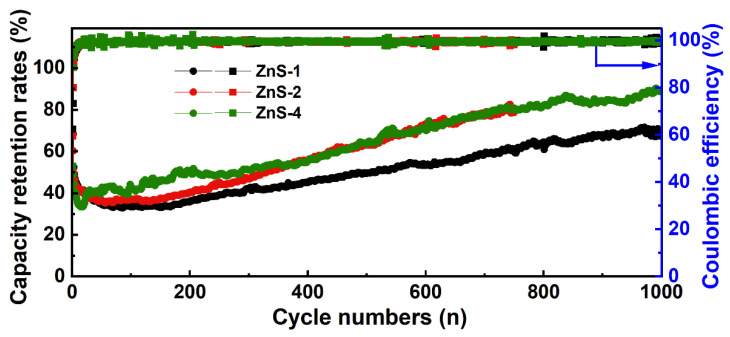
Capacity retention rates and Coulomb efficiency over multiple cycles for the other three composites (ZnS/C-1, ZnS/C-2, and ZnS/C-4).

Although the composite situation of ZnS and carbon in the other three ZnS/C materials is not as ideal as ZnS/C-3, we also conducted lithium storage performance tests on them to verify the effectiveness of the presence of the carbon framework, and the results are shown in Figure 10. In the cycle tests, the capacity also went through a process of initial decline followed by a gradual increase (corresponding to the capacity retention rate shown in Figure 11), which is consistent with that of the ZnS/C-3. At a current density of 0.5 A g^−1^, after cycling for hundreds or even a thousand times, the capacity could basically be maintained above 500 mAh g^−1^. Due to the formation of the SEI film and the irreversible intercalation of lithium ions, the initial CE of the three materials was relatively low. After a few cycles, it quickly increased to above 90%. In the subsequent cycling process, the CE remained close to 100% (Figure 11), indicating that all three materials exhibited good electrochemical reversibility. This also confirms the effectiveness of this general synthesis method for ZnS/C composites (used as anode materials for lithium storage) from different organic zinc salts.

## 3. Experimental Section

**Synthesis of ZnS/C-1:** In a ball milling jar (50 mL), 2 g of citric acid zinc (Macklin Reagents, Shanghai, China). All the reagents mentioned below are from this manufacturer), 0.955 g of thiourea, 3 g of sodium chloride, and 10 mL of ethanol were added, ball milled for 8 h (200 revolutions per minute), dried, and then transferred to a corundum boat. Then, the white powder was heated at 700 °C under a nitrogen atmosphere for 4 h (with a heating rate of 5 degrees per minute) and naturally cooled down to room temperature to obtain a black powder. The product was dispersed in 50 mL of diluted hydrochloric acid (0.1 mol L^−1^), stirred for two hours, vacuum filtered, washed with water and ethanol several times, and dried.

**Synthesis of ZnS/C-2:** The preparation method was the same as that of the ZnS/C-1, with the materials added being 2.135 g of glycine zinc, 0.914 g of thiourea, 3 g of sodium chloride, and 10 mL of ethanol.

**Preparation of ZnS/C-3:** Here, 2.435 g of lactate zinc, 0.914 g of thiourea, 3.4 g of sodium chloride, and 10 mL of ethanol were mixed, ball milled, and dried. Then, the white powder was pressed into several circular columns with a diameter of 15 mm (4–6 MPa for 20 s) and then heated to 700 °C for 4 h with the heating rate of 5 °C min^−1^ under N_2_ atmosphere. The subsequent treatment process was the same as that of ZnS/C-1.

**Preparation of ZnS/C-4:** Then, 2.5 g of gluconate zinc, 0.5 g of thiourea, and 3 g of sodium chloride were mixed, ball milled, and dried. The subsequent treatment process was the same as that of ZnS/C-3. The amount of hydrochloric acid used was changed to 25 mL.

### 3.1. Characterization

The phase analysis of the product was completed by X-ray diffraction (XRD) (D8 ADVANCE diffractometer, Bruker Co., Berlin, Germany). The morphology was observed by scanning electron microscopy (SEM) (Merlin Compact, Carl Zeiss AG, Oberkochen, Germany) and transmission electron microscopy (TEM) (Thermo Fischer, Talos F200x, Waltham, MA, USA). Electron diffraction, lattice, energy-dispersive spectroscopy, and element distribution were completed using a scanning electron microscope and a high-resolution transmission electron microscope (HRTEM) (Thermo Fischer, Talos F200x, Waltham, MA, USA) with an energy spectrometer attachment. The carbon content was determined by thermogravimetric analysis. Raman spectroscopy, specific surface area testing, and X-ray photoelectron spectroscopy (XPS) analysis were also performed.

### 3.2. Lithium Storage Performance Test

According to the ratio of 75:10:15, the prepared samples (ZnS/C), acetylene black, and PVDF (PVDF dissolved in NMP, prepared into a 3% mass concentration PVDF solution) were weighed, that is, 0.15 g of the prepared sample, 0.02 g of acetylene black, and 1 g of PVDF solution. The weighed materials were added to a mortar and ground for 40 min. If the paste was found to be sticky, an appropriate amount of NMP solvent could be added, and the materials could be ground for another 10 min. The obtained paste was applied to a copper foil. A 75-micron-thickness caliper was used. Then, the copper foil was transferred to a vacuum drying box, heated at 60 degrees for 8 h, and naturally cooled, and the copper foil was taken out and cut into a circle with a diameter of 12 mm. The load of the active material was about 0.7~1.0 mg cm^−2^. The circle was placed in a glove box, with a metal lithium sheet as the counter electrode, Celgard 2400 film as the separator, and 1 mol L^−1^ LiPF_6_ [in the mixture of EC:DEC (1:1 Vol% with 5% FEC)] as the electrolyte, to assemble a button battery. The assembled battery was left to stand overnight to ensure that the electrolyte could fully soak the electrode material and separator. Cyclic voltammetry (CV) was performed at a scan rate of 0.2 mV s^−1^ from 0.001 V to 3.0 V using a coin cell. Charge–discharge tests were performed between 0.01 and 3.0 V using a LANDCT2001A battery tester.

## 4. Conclusions

We used commercial thiourea and zinc lactate as raw materials and obtained a ZnS/C composite by heating at a nitrogen atmosphere for 4 h. At high temperature, the thermal decomposition process and the sulfidation process can be completed simultaneously, with ZnS nanoparticles being well encapsulated within the carbon framework. As an anode material for lithium-ion batteries, ZnS/C exhibits good reversible capacity and cycling stability. We expanded this synthesis method to obtain three other types of ZnS/C materials and tested their lithium storage performance, achieving satisfactory results. Furthermore, these four types of ZnS/C materials may also find applications in other electrochemical fields.

## Data Availability

The original contributions presented in this study are included in the article/Supplementary Materials. Further inquiries can be directed to the corresponding author(s).

## Supplementary Materials

The following supporting information can be downloaded at: https://www.mdpi.com/article/10.3390/molecules30040893/s1, Figure S1. SEM images of ZnS/C-1. Figure S2. SEM images of ZnS/C-2. Figure S3. SEM images of ZnS/C-3. Figure S4. SEM images of ZnS/C-4. Figure S5. HRTEM image of ZnS/C-3. Table S1. comparison between the ZnS/C-3 and reported ZnS/C-based materials.
