# Supplementary material for: Two-Dimensional Carbon Film-Supported ZnS Nanocomposites Obtained from Thermal Decomposition of Organic Zinc Salts and Sulfidation Reactions for Lithium Storage
Source: Molecules. 2025 Feb 14;30(4):893. doi: 10.3390/molecules30040893 (PMC11858112; doi:10.3390/molecules30040893)
Supplement: Supplementary file 1 [file molecules-30-00893-s001.zip › molecules-3395151-supplementary.pdf]

Supporting Information

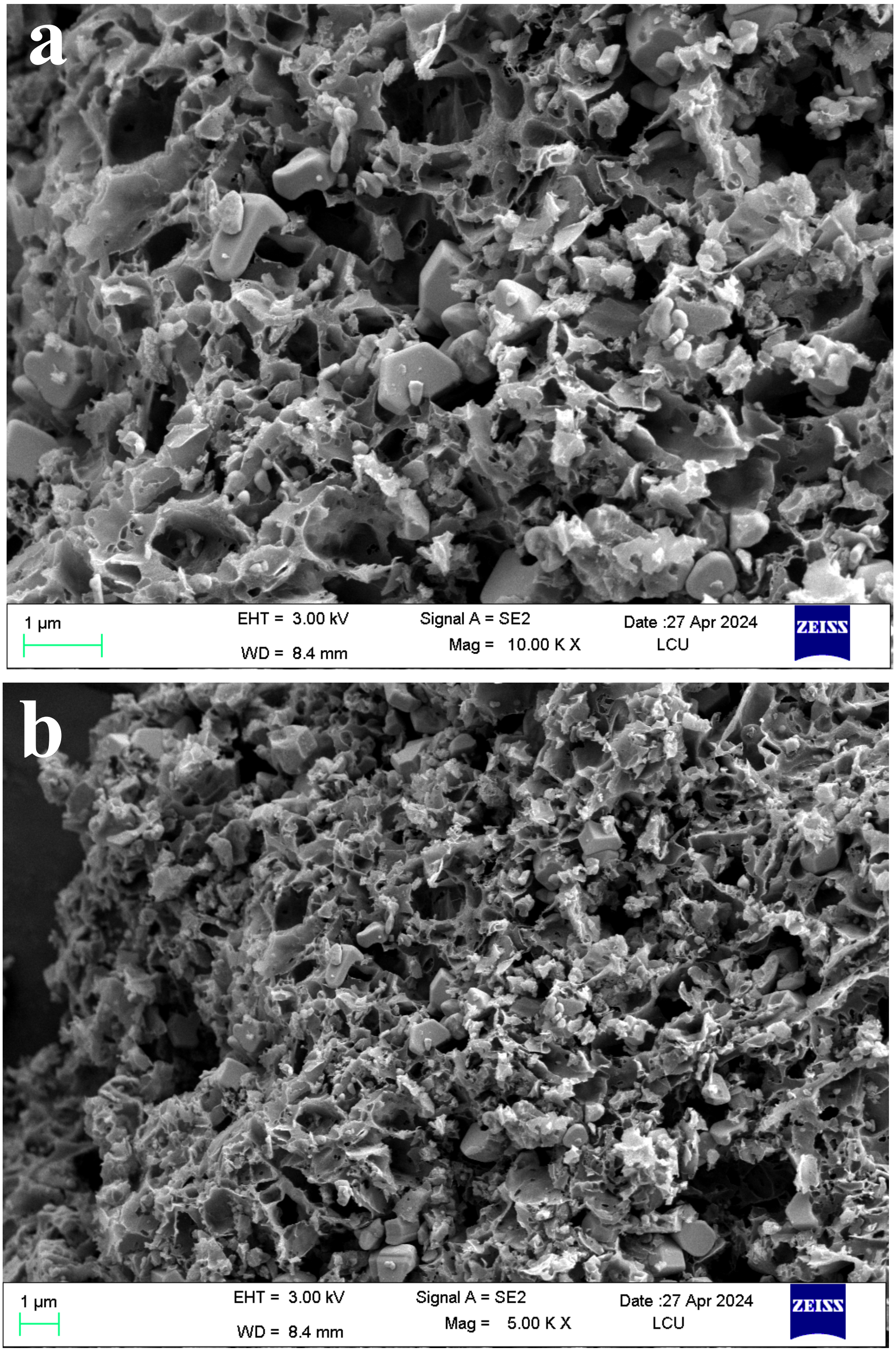

Figure S1 SEM images of ZnS/C-1

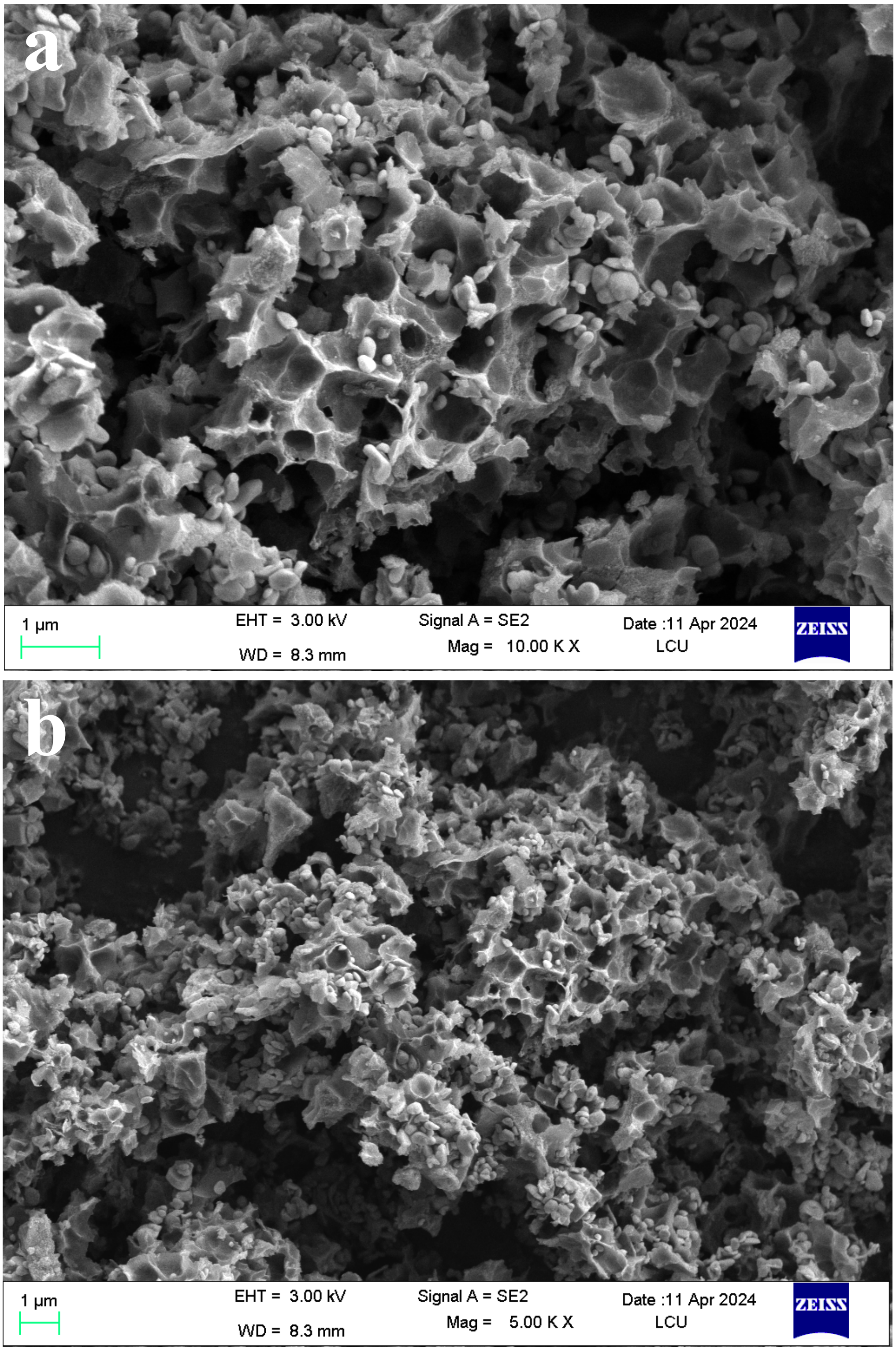

Figure S2 SEM images of ZnS/C-2

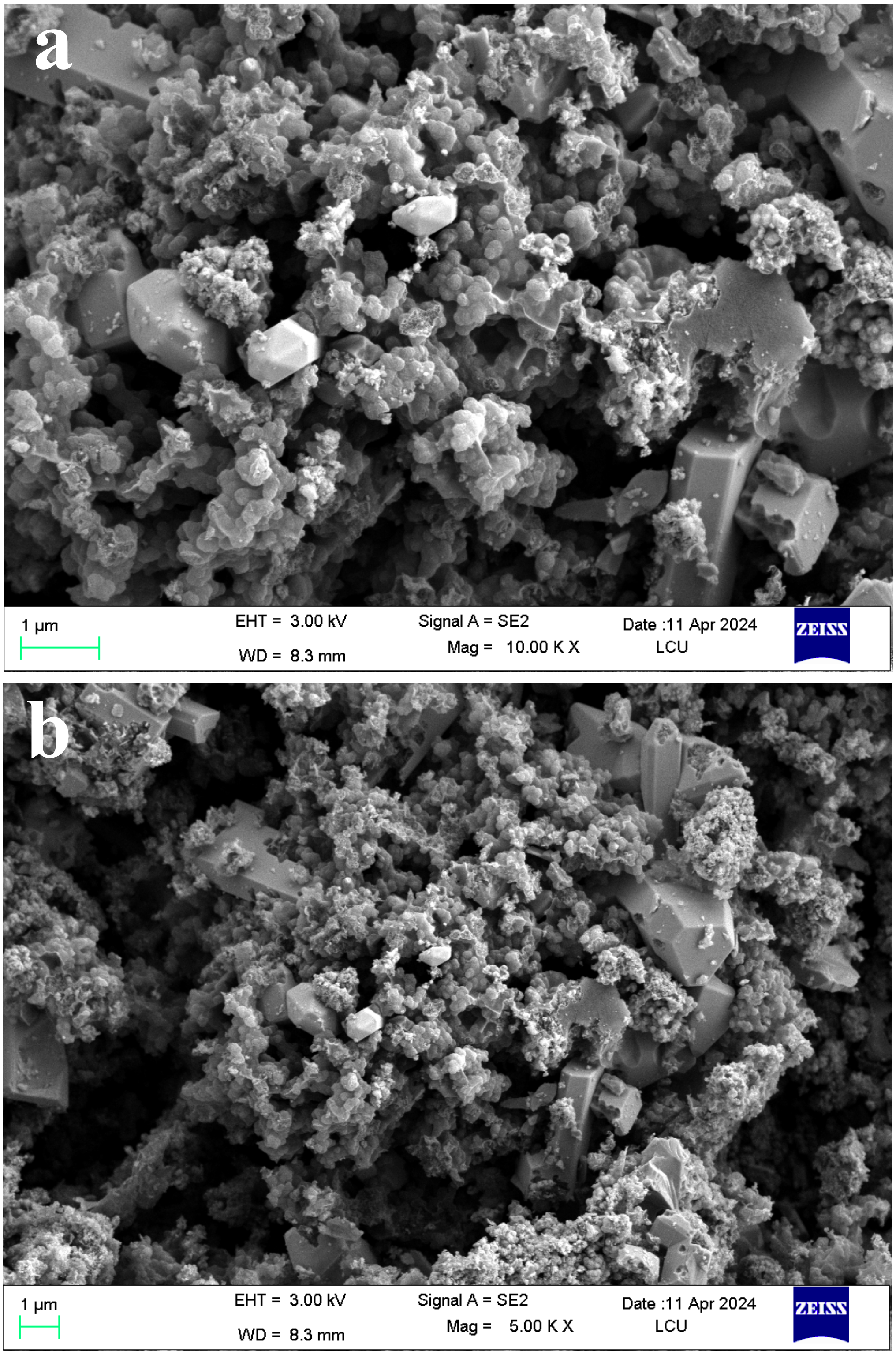

Figure S3 SEM images of ZnS/C-3

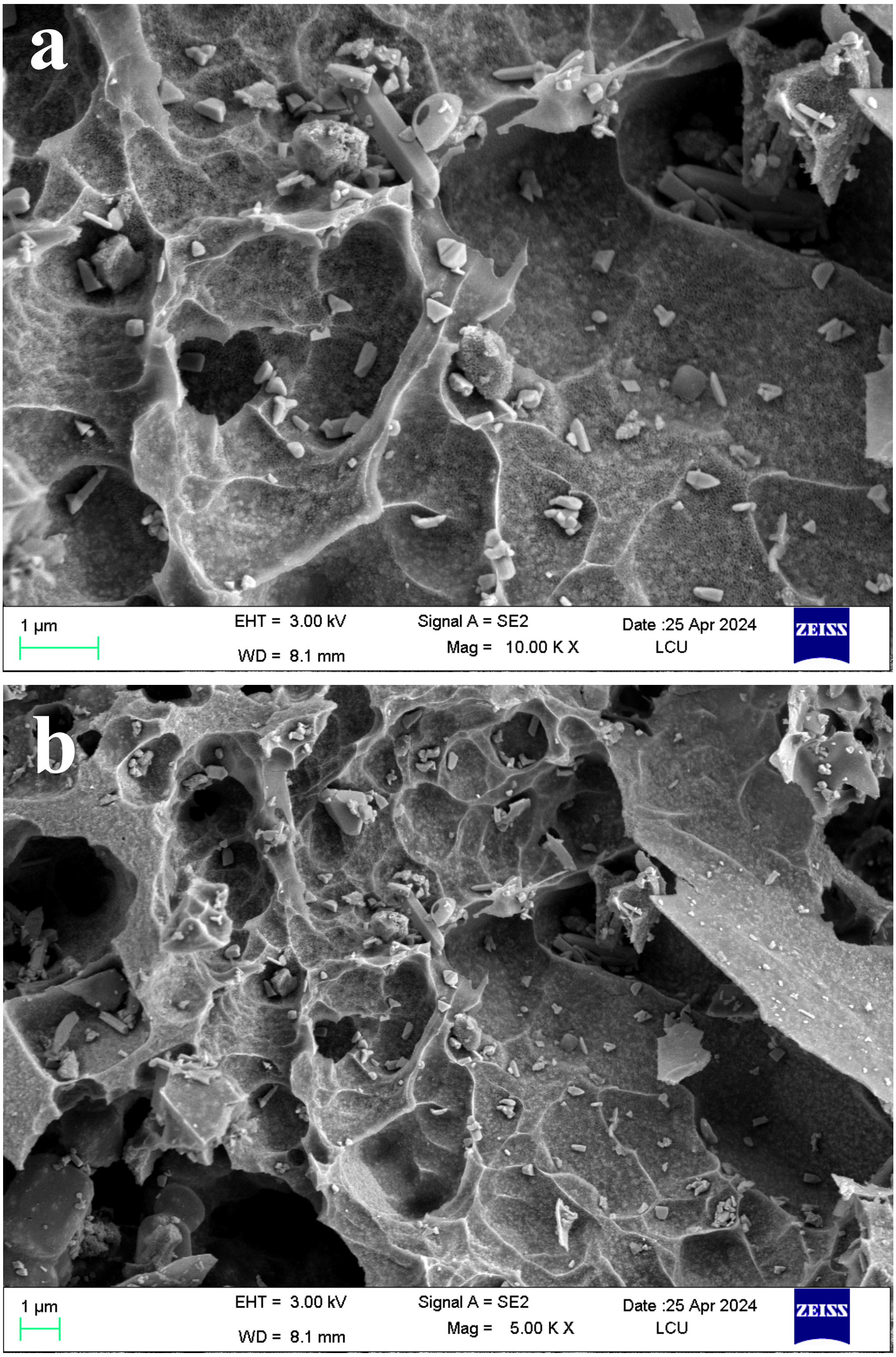

Figure S4 SEM images of ZnS/C-4

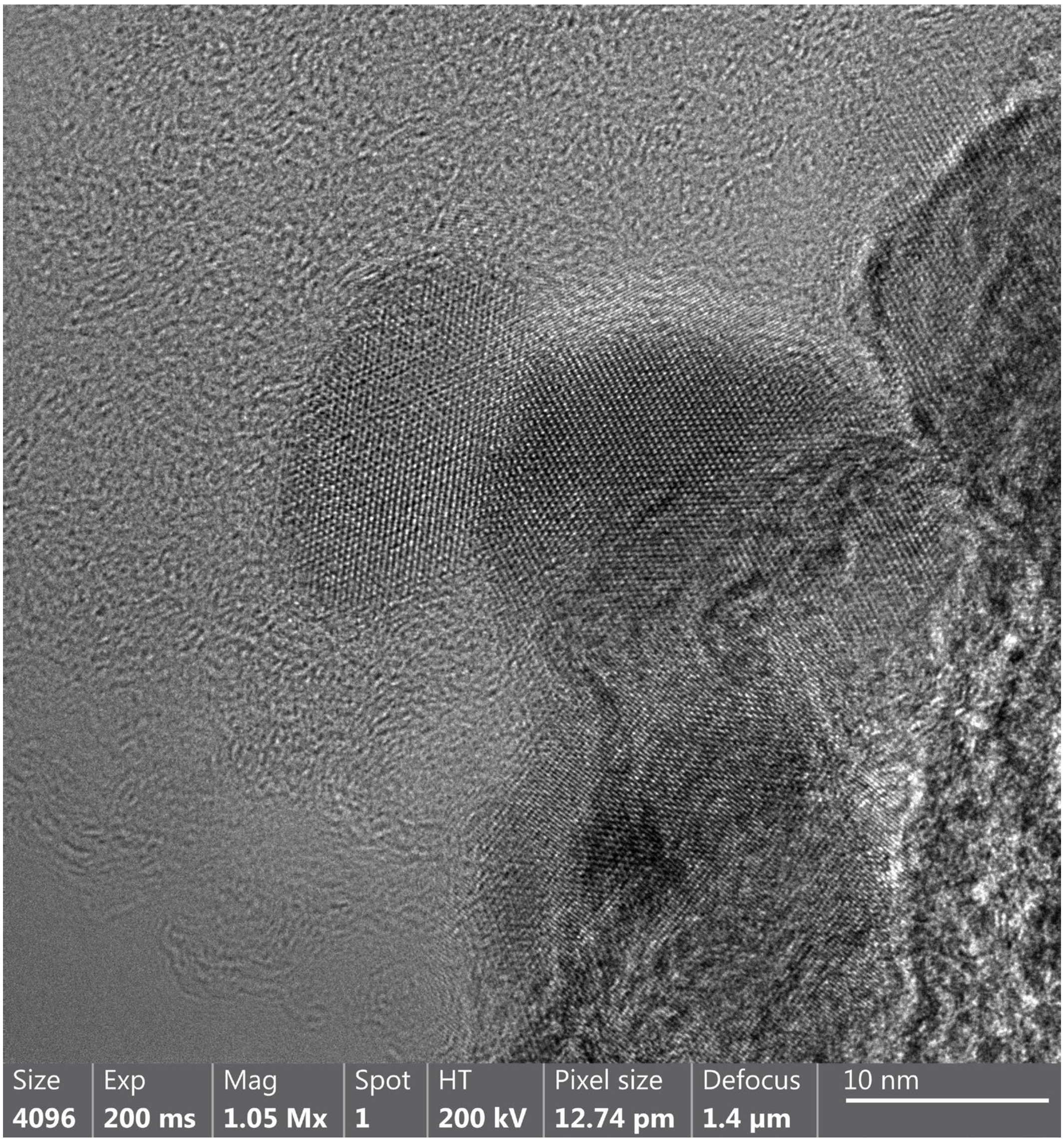

Figure S5 HRTEM image of ZnS/C-3

Table S1 comparison between the ZnS/C-3 and reported ZnS/C-based materials

Material	Capacity (mAh/g)	Cycle number	Rate (A/g)	Ref.
3D ZnS@CN	605.7	400	0.5	[9]
ZnS/NS-CN	291.6	1000	5	[10]
ZnS/NC	500	1000	2	[11]
CC-ZnS/CNT	333	4000	2	[12]
ZnS/ZnO-C	535.6	200	1	[14]
ZnO/ZnS@N-C	386.6	400	1	[15]
ZnS/C-3	749	1000	0.5	This work
ZnS/C-3	563.6	1350	1	This work
